# Constant light enhances synchrony among circadian clock cells and promotes behavioral rhythms in VPAC_2_-signaling deficient mice

**DOI:** 10.1038/srep14044

**Published:** 2015-09-15

**Authors:** Alun T.L. Hughes, Cara. L. Croft, Rayna E. Samuels, Jihwan Myung, Toru Takumi, Hugh D. Piggins

**Affiliations:** 1 Faculty of Life Sciences, University of Manchester, Manchester, UK; 2 RIKEN Brain Science Institute, Wako, Saitama, Japan

## Abstract

Individual neurons in the suprachiasmatic nuclei (SCN) contain an intracellular molecular clock and use intercellular signaling to synchronize their timekeeping activities so that the SCN can coordinate brain physiology and behavior. The neuropeptide vasoactive intestinal polypeptide (VIP) and its VPAC_2_ receptor form a key component of intercellular signaling systems in the SCN and critically control cellular coupling. Targeted mutations in either the intracellular clock or intercellular neuropeptide signaling mechanisms, such as VIP-VPAC_2_ signaling, can lead to desynchronization of SCN neuronal clocks and loss of behavioral rhythms. An important goal in chronobiology is to develop interventions to correct deficiencies in circadian timekeeping. Here we show that extended exposure to constant light promotes synchrony among SCN clock cells and the expression of ~24 h rhythms in behavior in mice in which intercellular signaling is disrupted through loss of VIP-VPAC_2_ signaling. This study highlights the importance of SCN synchrony for the expression of rhythms in behavior and reveals how non-invasive manipulations in the external environment can be used to overcome neurochemical communication deficits in this important brain system.

In mammals, the dominant light-entrainable circadian pacemaker is contained in the hypothalamic suprachiasmatic nuclei (SCN). Individual SCN neurons function as autonomous circadian oscillators, expressing ~24 h oscillations in core clock genes/proteins, such as *per1-2/*PER1-2^1^. Such intracellular molecular rhythms can be visualized over several cycles *in vitro* in individual cells of living SCN brain slices prepared from mice in which an enhanced destabilized green fluorescent protein (eGFP) reports the activity of the *per1* promoter (*per1*::eGFP mice^2^). Rhythmic expression of clock genes does not itself define SCN timekeeping; these cell-autonomous oscillators must synchronize their activities to produce global SCN output of sufficient amplitude and coherence to organize circadian rhythms in whole animal physiology and behavior^3^. Intra-SCN signaling utilizes a range of neurotransmitters including arginine vasopressin (AVP), vasoactive intestinal polypeptide (VIP), gastrin-releasing peptide (GRP) glutamate and γ-aminobutyric acid (GABA). Both VIP and its receptor, VPAC_2_, are expressed by SCN neurons^4^,^5^ and intercellular communication via VIP-VPAC_2_ signaling plays a key role in the coupling of SCN cellular timekeepers^6^,^7^. For example, mice lacking VIP or VPAC_2_ expression (*Vip*^*−/−*^ and *Vipr2*^*−/−*^, respectively) exhibit pronounced abnormalities in the timing of their behavior and physiology, and SCN expression of clock genes/proteins is greatly diminished^8^,^9^,^10^,^11^. Indeed, in comparison to SCN from behaviorally rhythmic wild-type (WT) *per1*::eGFP mice, single cell oscillators in the *Vipr2*^*−/−*^ × *per1*::eGFP SCN are fewer in number, oscillate at lower amplitude, and are less well synchronized^7^. Therefore, circadian deficits observed *in vivo* can be accurately mapped to *ex vivo* assessments of SCN clock cell activity.

Animals with functional deficits in the intracellular molecular clockworks can express aberrant circadian behavior in constant darkness (DD)^12^, with SCN rhythms in clock genes typically blunted or disrupted^13^,^14^,^15^. Intriguingly, behavioral and SCN molecular rhythms can be restored in some such models by maintaining them in constant light (LL)^16^,^17^,^18^. Light information is relayed to the SCN via the retinohypothalamic tract (RHT), using glutamate and pituitary adenylate cyclase-activating polypeptide (PACAP) as neurotransmitters^19^,^20^, and induces expression of clock genes including *per1*^21^. It is possible that LL provides a tonic excitatory drive onto the SCN thereby circumventing intracellular molecular lesions^16^, though it is unclear if LL can also restore circadian activities in mice with intercellular signaling defects. Since *Vipr2*^*−/−*^ mice express all the known clock genes, albeit at diminished levels^8^, and because the light input pathway in *Vipr2*^*−/−*^ mice is intact^22^,^23^,^24^,^25^, we investigated whether LL could rescue behavioral and SCN cellular rhythms in this model of a circadian system weakened through impairment of the key intercellular synchronizing pathway. We report that extended exposure to LL promotes behavioral rhythms and SCN intercellular synchrony in *Vipr2*^*−/−*^ mice. This indicates that manipulations based on non-invasive lighting strategies can be effective to improve circadian competence and highlights the plastic nature of SCN circadian function.

## Results

### Differential Effects of Constant Light on Wheel-Running Behavior in WT and *Vipr2* ^*−/−*^ Mice

**Figure 1:**
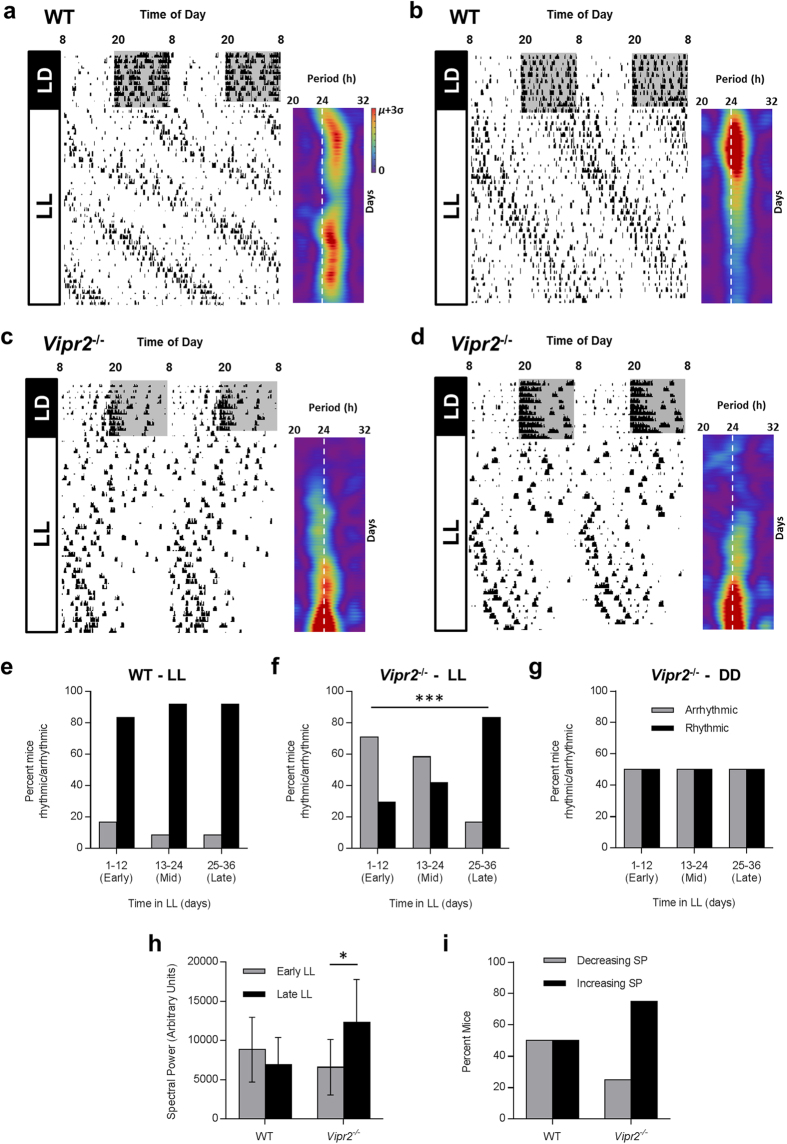
Exposure to LL improves the expression of circadian behavior in *Vipr2*^*−/−*^ mice. Actograms showing wheel-running behavior of WT and *Vipr2*^*−/−*^ mice in LD and LL, with complimentary FFT spectrograms for the LL portion of activity data (**a**–**d**). Summary histograms show rhythmicity of WT and *Vipr2*^*−/−*^ mice in LL (**e**,**f**) and subsequent DD for a subset of mice (**g**, also see Fig. 2); rhythm strength (FFT spectral power) in early vs late LL (**h**) and the percentage of mice that showed increasing and decreasing rhythm strength over time in LL (**i**). After initial disruption, LL improves circadian behavior in *Vipr2*^*−/−*^ mice. For baseline comparison, DD rhythmicity data are presented in panel (**g**) for the subset of 12 *Vipr2*^*−/−*^ mice that were exposed to LL-DD-LL conditions (actograms shown in Fig. 2). Actograms show behavior of individual animals and are double-plotted with 2 consecutive days’ data on each line. Spectrograms are presented aligned horizontally with the corresponding behavioral data and vertical white dotted lines show the 24 h period mark. The color scale is normalized between 0 spectral power (purple) and mean spectral power plus 3 standard deviations (μ + 3σ; red). Color scale for spectrograms in (**b**–**d**) is as shown in (**a**). Gray shading on actograms indicates darkness. Histogram legends for (**e**,**f**) are as shown in (**g**). Panel **f** shows a significant increase in the proportion of rhythmic *Vipr2*^*−/−*^ mice over time in LL (*p*<0.001; Fisher’s Exact Test with Freeman-Halton extension), an effect which is absent for both *Vipr2*^*−/−*^ mice in DD (**g**) and WT mice in LL (**e**). Panel **h** shows a significant increase in rhythm strength (FFT spectral power) of *Vipr2*^*−/−*^ mice in late LL vs early LL (*p*<0.01; paired *t*-Test). Panel **i** provides a visual illustration that rhythm strength increases over time in LL in the majority of *Vipr2*^*−/−*^ mice, but not WT mice. * *p*<0.05; *** *p*<0.001.

Both WT and *Vipr2*^*−/−*^ mice confined the majority of intense wheel-running activity to the dark phase of the LD cycle. On transfer to LL, WT mice behaved in a manner consistent with previous descriptions^26^,^27^,^28^, exhibiting a large phase delay in locomotor activity (~5 h), and suppression of wheel-running compared to LD (~110 revs/h in LL vs. ~400 rev/h in LD; *p* = 0.001; *t*-Test; n = 12; Fig. 1a,b). Initial disruption of rhythmicity, lasting ~2–3 days, was followed for the majority (11 of 12) of individuals by more robust rhythms that persisted throughout the 36 days of LL examined here. Unlike DD behavior, where arrhythmicity in WT mice is uncommon and unexpected^24^,^29^,^30^, one WT mouse failed to express identifiable circadian rhythms in behavior in LL demonstrating, as has previously been reported, that LL can be disruptive to WT circadian rhythms in locomotor activity. Over the time course of LL examined here, we observed no significant change in the percentage of rhythmic WT mice (*p* = 0.677; Fisher’s Exact Test with Freeman-Halton extension; n = 12; Fig. 1e;[Fig f2][Fig f3] early vs. mid vs. late LL; though also see Fig. 4a) and no significant change in rhythm strength (*p* = 0.25; paired *t*-Test of FFT spectral power in early vs late LL; n = 12; Fig. 1h). Indeed, 50% of WT individuals exhibited increased spectral power in late LL vs early, while 50% exhibited a decrease (Fig. 1i). As is common in LL, rhythmic WT mice expressed a mean period that was substantially longer (25.03 ± 0.11 h; see Fig. 1a,b) than is observed for this strain in DD^7^,^31^.

**Figure 2 f2:**
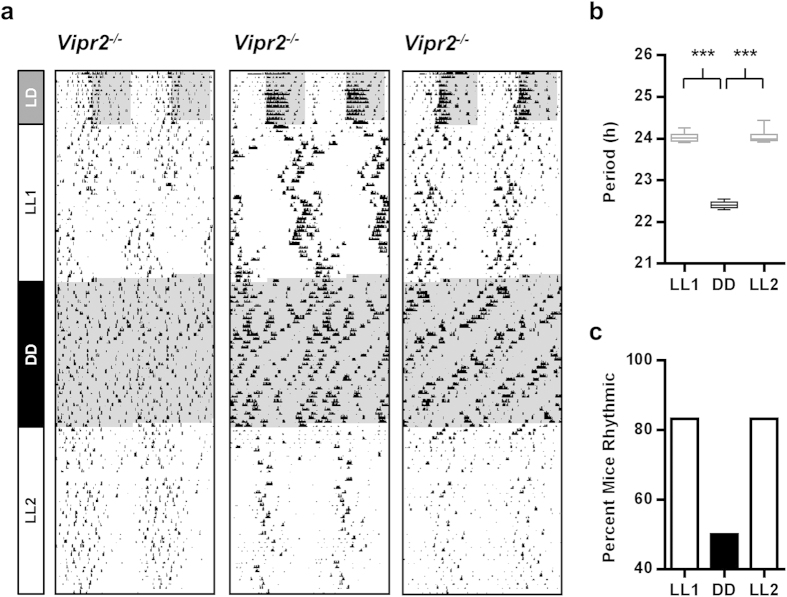
LL-induced improvement in circadian behavior of *Vipr2*^−/−^ mice depends on continued exposure to LL. Actograms showing wheel-running behavior of *Vipr2*^*−/−*^ mice housed under LL conditions for 36 days, then transferred into DD for 36 days, before a second 36 day exposure to LL (**a**). On transfer to DD, rhythmic *Vipr2*^*−/−*^ mice do not sustain the ~24 h rhythms expressed in LL and instead become arrhythmic or show shorter period (~22.4 h) rhythms in wheel-running (also see 24). When subsequently re-exposed to LL, *Vipr2*^*−/−*^ mice rapidly alter wheel-running to once again show overt rhythmicity and the characteristic longer period (~24 h) expressed in LL. Actogram plotting and shading as in Fig. 1. Panels (**b**,**c**) show a box and whisker plot of period and histogram of rhythmicity, respectively, for LL1, DD and LL2, assessed over the last 12 days of each epoch. Whiskers indicate the maximum and minimum range of period data points. ***p < 0.001.

The majority of *Vipr2*^*−/−*^ individuals exhibited overtly disrupted rhythms on release into LL, though unlike WT mice, this disruption persisted substantially longer than the first 2–3 days in LL (Fig. 1c,d). Indeed, while typically ~50% of *Vipr2*^*−/−*^ mice generate robust rhythms in locomotor behavior in DD (Figs 1g and 2 and see^24^), only ~30% of *Vipr2*^*−/−*^ mice (7 of 24) exhibited identifiable circadian rhythms in wheel-running during early LL (Fig. 1f). With increasing duration in LL, however, the percentage of rhythmic individuals significantly increased, reaching 83% (20 of 24 individuals) by late LL (Fig. 1f; *p* = 0.00032, Fisher’s Exact Test with Freeman-Halton extension; n = 24). Consistent with this, rhythm strength of *Vipr2*^*−/−*^ mice significantly increased over time in LL (Fig. 1h; *p* = 0.040; paired *t*-Test of FFT spectral power in early vs late LL; n = 24), an increase observed in 75% of *Vipr2*^*−/−*^ individuals (Fig. 1i). As such, the effect of LL on *Vipr2*^*−/−*^ locomotor rhythms was time-dependent, with initial disruption of rhythmicity in the short term followed by a rhythm enhancing effect after extended exposure.

Rhythmic *Vipr2*^*−/−*^ mice (n = 20) expressed a mean period of 24.29 ± 0.08 h in LL, significantly shorter than WT mice under these conditions (*p* = 0.000009; n = 11 WT and 20 *Vipr2*^*−/−*^). Once rhythmic, the period of *Vipr2*^*−/−*^ mice remained consistent throughout the duration examined (Fig. 1c,d) and was longer than has been observed for this strain in DD^24^,^32^ (and see Fig. 2). Wheel-running activity (rev/h) of *Vipr2*^*−/−*^ mice did not significantly reduce on transfer from LD to LL (173 ± 23 vs. 135 ± 20 rev/h, respectively; *p* = 0.35; n = 24; Fig. 1c,d), and this parameter of *Vipr2*^*−/−*^ wheel activity under LL was not significantly different to that of WT animals under LL (*p* = 0.50; n = 12 WT and 24 *Vipr2*^*−/−*^).

Thus, exposure to LL has a disruptive effect on the expression of WT behavioral rhythms but induces a time-related enhancement of rhythmicity in *Vipr2*^*−/−*^ mice after extended durations.

### LL-induced Improvements in Behavioral Rhythmicity Are Not Sustained in the Absence of Constant Light

To test whether LL-induced improvements in behavioral rhythmicity persisted in the subsequent absence of light, following the initial 36 days of LL (LL1) a subgroup of 12 *Vipr2*^*−/−*^ mice were transferred to DD for 36 days. The rhythm characteristics of this subset were representative of the whole *Vipr2*^*−/−*^ group examined in LL1. On transfer into DD, *Vipr2*^*−/−*^ mice rapidly reverted to behavioral phenotypes well-documented for this genotype under DD conditions^24^,^32^,^33^; a continuum of phenotypes was observed across individuals, spanning arrhythmicity through to rhythmic with a short period (Fig. 2a). Whilst 83% of this cohort had expressed a circadian rhythm in behavior at the end of LL1, only 50% were rhythmic during DD, and the period of these rhythms, where present, was reduced by ~1.6 h from near 24 h to ~22.4 h (24.03 ± 0.04 h for this subset in LL1 (n = 10) to 22.41 ± 0.04 h in DD (n = 6); *p* < 0.001; Fig. 2). The rhythmicity of these mice did not overtly change across the duration DD examined here (Figs 1g and 2a). This subgroup of *Vipr2*^*−/−*^ mice were subsequently returned to LL for a further 36 days (LL2) during which each individual expressed a locomotor phenotype consistent with its behavior during LL1 (83% (10 of 12) rhythmic in LL2, mean period 24.05 ± 0.05 h; Fig. 2). *Vipr2*^*−/−*^ mice re^*-*^ exposed to this second epoch of LL tended to regain the ‘LL-like’ behavioral phenotype more rapidly than during the original LL exposure (e.g. Fig. 2a).

### Intercellular Synchrony in the SCN Correlates with Behavioral Rhythmicity

**Figure 3 f3:**
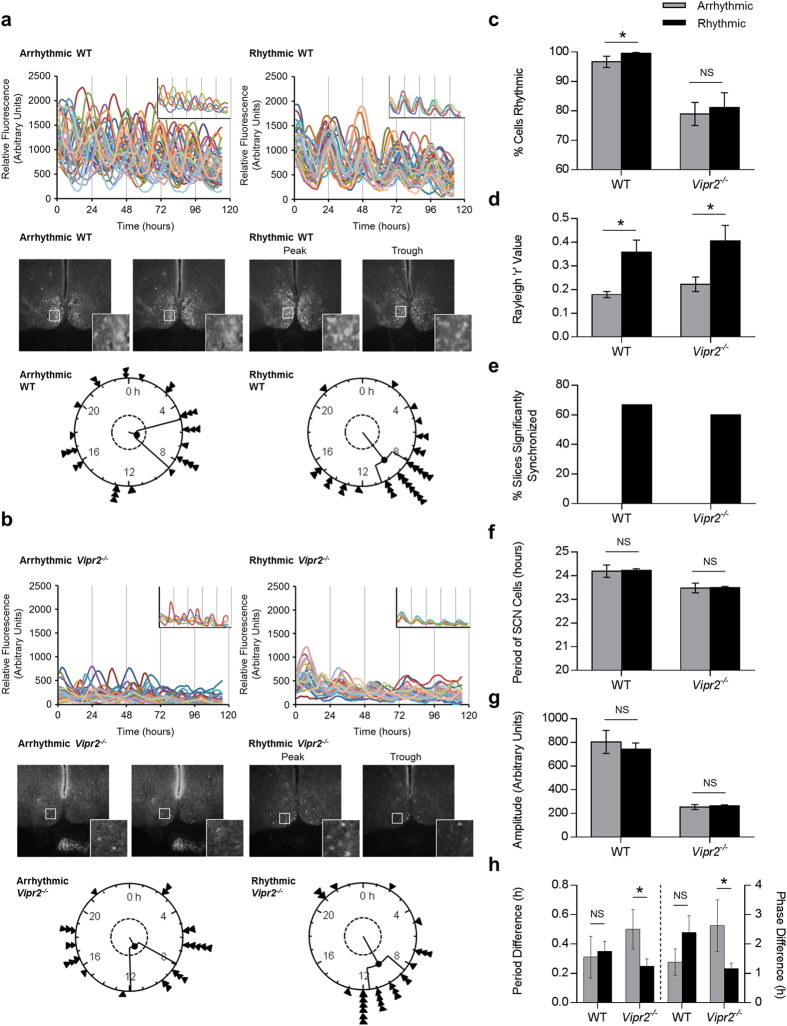
Behavioral rhythmicity in WT and *Vipr2*^*−/−*^ mice is associated with increased intercellular synchrony in the SCN. a-b (upper panels). Composite rhythm plots show *per1*::eGFP profiles for 30 individual SCN cells in two brain slices from each genotype. Insets show a reduced selection of cells to aid visualization of synchrony and highlight differences in synchrony between cells within slices. (**a**–**b**) (**middle panels**): Photomicrographs showing peak and trough expression of *per1*::eGFP for rhythmic slices and representative images from equivalent timepoints for arrhythmic slices. Insets show the regions indicated by white boxes at higher magnification (note synchronized peak and trough expression for SCNs from rhythmic mice and lack of coherent day-night differences for SCNs from arrhythmic mice). (**a**,**b**) (**lower panels**): Rayleigh plots showing time of peak *per1*::eGFP expression of rhythmic SCN cells analyzed within single slice cultures. Arrow heads indicate the time of peak fluorescence for individual cells, the length of the central line indicates the degree of synchrony between the times of peak expression of individual cells (quantified as Rayleigh R; longer line indicates greater synchrony) and the inner broken ring shows the threshold for statistical significance of synchrony. (**c**–**h**): Histograms describing analyzed rhythm parameters for *per1*::eGFP expression in slices from rhythmic and arrhythmic WT and *Vipr2*^*−/−*^ mice. Key for panels **d**–**h** is as shown in panel **c**. Behavioral rhythmicity was assessed during LL for the last 10–14 days before cull and data are presented as a comparison between the SCN parameters of mice classified as behaviorally rhythmic and arrhythmic, irrespective of time in LL (which is considered in Fig. 4). Panel (**h**) shows period difference and phase difference between dorsal and ventral parts of the SCN in cultures from behaviorally rhythmic and arrhythmic WT and *Vipr2*^*−/−*^ mice. Across both genotypes, most SCN cultures from behaviorally rhythmic mice were synchronized (**d**,**e**), while no SCN cultures from behaviorally arrhythmic animals expressed significantly synchronized cellular rhythms (**e**). SCN slices from behaviorally rhythmic WT mice contained significantly more rhythmic cells (**c**), whereas SCN slices from behaviorally rhythmic *Vipr2*^*−/−*^ mice exhibited reduced dorsal-ventral regional heterogeneity in period and phase (**h**).

Given the time-dependent effect of LL on *Vipr2*^*−/−*^ behavior, we next assessed the correlation of LL-induced behavioral changes with *per1*::eGFP expression in the SCN. A separate cohort of behaviorally phenotyped WT and *Vipr2*^*−/−*^ mice were culled at randomly assigned timepoints after increasing durations of LL and, using confocal microscopy, we imaged *per1*::eGFP expression in live SCN-containing brain slice cultures from each individual (Fig. 3).

**Figure 4:**
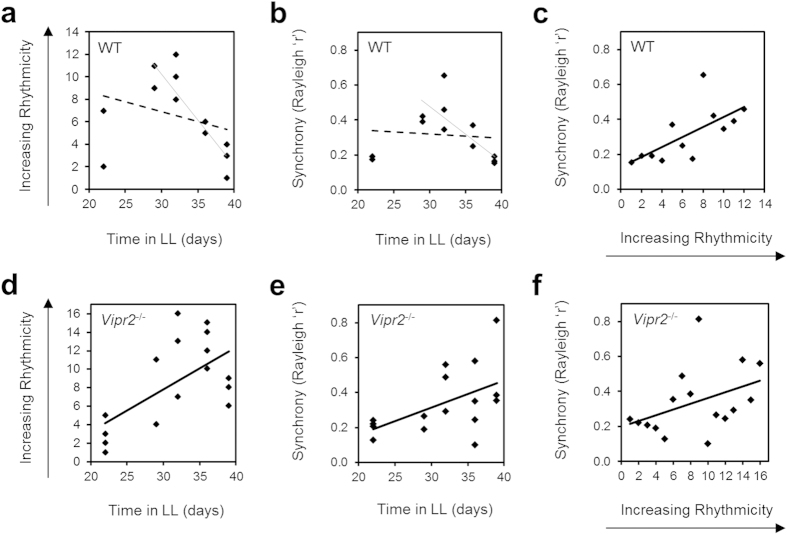
Relationships between time in LL, behavioral rhythmicity and SCN cellular synchrony for WT and *Vipr2*^−/−^ mice. *Vipr2*^*−/−*^ data describe a three-way positive correlation between increasing time in LL, greater behavioral rhythmicity and increasing SCN cellular synchrony (**d**–**f**). For WT mice, behavioral rhythmicity positively correlates with SCN cellular synchrony (**c**). Conversely, the relationships of both of these parameters with time in LL for WT mice are poorly fit by linear trends (broken lines on panels **a**,**b**) but ultimately result in a progressive decrease in both rhythmicity (**a**) and synchrony (**b**) after extended (>29 days) durations of LL (light unbroken lines added for visual reference; though see main text for statistical assessment of these subsets of data for >29 days in LL). Data in panels **c**–**f** are shown fitted with linear trend lines (heavy unbroken lines) that provided high goodness of fit and illustrate a linear relationship of ranked data in each case (all data sets in panels (**c**–**f**) show significant correlations; Spearman’s *rho* = 0.755 (*p* = 0.0025) (**c**), 0.522 (*p* = 0.038) (**d**), 0.6 (*p* = 0.007) (**e**) and 0.512 (*p* = 0.0215) (**f**)).

Mice used in this part of the study showed the same trends in behavioral rhythmicity as those in the initial behavioral examinations presented previously. Indeed, ranking all *Vipr2*^*−/−*^ mice used for confocal imaging in order of behavioral rhythmicity at the time of cull revealed a significant positive correlation between time in LL and ranked behavioral rhythmicity (Spearman’s *rho* value 0.522; *p* = 0.038; *R*^2^ = 0.387; n = 16; Fig. 4d). A linear trend line poorly fitted the data for WT time in LL plotted against behavioral rhythmicity rank (*R*^2^ = 0.085; Fig. 4a), and these data were not significantly correlated (Spearman’s *rho* = −0.442; *p* = 0.15; n = 12). However, visual interpretation of the data revealed that with extended durations of LL (>29 days), rhythmicity consistently decreased in WT mice, an association that did reach statistical significance for this subset of animals (Spearman’s *rho* = −0.813; *p* = 0.005; n = 10; Fig. 4a).

To assess the relationship between behavioral rhythmicity and circadian function in the SCN at single cell level, we divided *per1*::eGFP expression data for each genotype into 2 groups; individuals behaviorally rhythmic at the time of cull (n = 9 WT; n = 10 *Vipr2*^*−/−*^), and individuals behaviorally arrhythmic at cull (n = 3 WT; n = 6 *Vipr2*^*−/−*^). The time of cull for each individual was assigned prior to the experiment commencing and therefore was not influenced by the degree of behavioral rhythmicity of the animals. SCN slices from behaviorally rhythmic WT mice contained a significantly higher percentage of rhythmic individual cells than slices from behaviorally arrhythmic conspecifics (99.6 ± 0.4% vs. 96.7 ± 1.9%; *p* = 0.016; Fig. 3a,c). Intriguingly, however, we found no significant difference in the percentage of rhythmic cells between slices from behaviorally rhythmic and arrhythmic *Vipr2*^*−/−*^ mice (both ~80% of cells rhythmic; *p* = 0.380; Fig. 3b,c).

Crucially, this intergenotype difference in the relationship of behavioral and SCN cellular rhythmicity was not seen for synchrony between cellular rhythms in the SCN; we observed significantly greater intercellular synchrony within slices from behaviorally rhythmic WT and *Vipr2*^*−/−*^ mice than in slices from arrhythmic conspecifics (0.36 ± 0.05 vs. 0.18 ± 0.01; *p* = 0.0039 [synchrony in slices from rhythmic WT mice vs. arrhythmic WT mice, respectively]; and 0.41 ± 0.07 vs. 0.22 ± 0.03; *p* = 0.0284 [slices from rhythmic vs. arrhythmic *Vipr2*^*−/−*^ animals]; Rayleigh R values; Fig. 3a,b,d). Further, while 67% of slices from behaviorally rhythmic WT animals were significantly synchronized, and 60% of slices from behaviorally rhythmic *Vipr2*^*−/−*^ mice, no slices from behaviorally arrhythmic mice of either genotype exhibited significant intercellular synchrony (Fig. 3a,b,e). We found no significant differences in the mean period or amplitude of cells between slices from behaviorally rhythmic and arrhythmic individuals for either WT or *Vipr2*^*−/−*^ mice (all *p* < 0.05) though both the period and amplitude of *Vipr2*^*−/−*^ oscillations were consistently lower than those of WT oscillations, regardless of behavioral rhythmicity (all *p* < 0.001; Fig. 3f,g).

As regional heterogeneity in period and phase within the SCN have previously been associated with the maintenance of circadian synchrony^34^, we next assessed differences in these parameters between the dorsal and ventral regions of the SCN in behaviorally rhythmic and arrhythmic mice of both genotypes. In WT SCN we found no significant changes in dorsal-ventral period or phase heterogeneity between behaviorally rhythmic and arrhythmic animals (Fig. 3h). In *Vipr2*^*−/−*^ SCN, however, the differences in both period and phase between dorsal and ventral regions were significantly smaller (as well as less variable; see smaller SEM) in slices from behaviorally rhythmic mice (Fig. 3h), a characteristic consistent with improved cellular synchrony in behaviorally rhythmic animals.

Finally, to better describe the genotype-dependent and -independent aspects of the relationships between behavioral rhythmicity, SCN synchrony and time in LL, we calculated Spearman’s Rank Correlation Coefficients for these parameters using behavioral data ranked in order of rhythmicity. For both WT (n = 12) and *Vipr2*^*−/−*^ (n = 16) mice we found a significant correlation between ranked behavioral rhythmicity and intercellular synchrony (*p* = 0.0025 [Spearman *rho* = 0.755] for WT and *p* = 0.0215 [0.512] *Vipr2*^*−/−*^) and that a linear trend line fit the data well (*R*^2^ = 0.462 and 0.168, respectively; Fig. 4c,f). Consistent with this, and with the positive correlation between time in LL and behavioral rhythmicity in *Vipr2*^*−/−*^ mice (Fig. 4d), we also found a significant positive relationship between time in LL and SCN intercellular synchrony in *Vipr2*^*−/−*^ mice (*p* = 0.007; *rho* = 0.6; Fig. 4e). In WT mice however, as we observed for the relationship between time in LL and behavioral rhythmicity (see above; Fig. 4a), time in LL was not significantly correlated with SCN synchrony (Spearman’s *rho* = −0.374) and a linear trend line poorly fitted the data (*R*^*2*^ = 0.010; Fig. 4b). Similarly to time in LL vs. behavioral rhythmicity for WT mice however, visual interpretation of the time in LL vs. cellular synchrony plot for WT mice suggested progressively decreasing synchrony later in LL at extended (>29 days) durations of LL; we found a significant correlation between these parameters in WT mice when we assessed this >29 day in LL subset of animals only (Spearman’s *rho* = −0.875; *p* = 0.001; n = 10; Fig. 4b).

## Discussion

Here we show that longer term exposure to constant light, a non-invasive environmental manipulation, promotes ~24 h behavioral rhythmicity and stable, synchronized cellular rhythms in the master circadian pacemaker of mice with an intercellular signaling deficit. This is in marked contrast to the disruptive and potentially detrimental influence of the same stimulus, constant light exposure, on circadian rhythms in animals with an intact circadian timing system. These findings provide novel insight into the restoration of circadian rhythmicity at behavioral and single cell levels in mice deficient in signaling via the VPAC_2_ receptor; a major conduit for SCN intercellular communication.

LL differentially altered the expression of behavioral rhythmicity in individual WT and *Vipr2*^*−/−*^ mice. To identify potential factors underpinning the circadian profile of behavior, we assessed synchrony among SCN clock cells of rhythmic and arrhythmic animals of both genotypes. Compared with behaviorally rhythmic individuals, synchrony among *per1*::eGFP SCN neurons of behaviorally arrhythmic mice was reduced, regardless of genotype. Indeed in both genotypes, statistically significant intercellular synchrony was absent in slices from all behaviorally arrhythmic individuals. Importantly, the degree of synchrony among SCN clock cells from behaviorally rhythmic animals did not differ between WT and *Vipr2*^*−/−*^ mice, despite a lower proportion of rhythmic individual cells in the *Vipr2*^*−/−*^ SCN. In *Vipr2*^*−/−*^ mice we found that, for both period and phase, the regional differences between dorsal and ventral areas of the SCN were reduced in rhythmic individuals. As such, across both genotypes, greater behavioral rhythmicity was associated with increased intra-SCN synchrony, but different features of the circadian architecture of the SCN are permissive of this effect between mice with intact and disrupted circadian timing systems. Notably, both temporal and spatial organization of SCN circadian function have been implicated in the generation and maintenance of robust tissue level oscillations^34^,^35^,^36^,^37^,^38^. Indeed, oscillations at the single-cell level have been reported to cluster into dorsal and ventral SCN subregions, with a smaller period difference between these subregions associated with greater cellular synchrony^38^. These data are consistent with the reduced dorsal-ventral SCN period difference in behaviorally rhythmic *Vipr2*^*−/−*^ mice under LL and the increased SCN cellular synchrony observed for these mice, as well as their longer free-running behavioral periods.

A previous report of restored behavioral rhythmicity in mice bearing intracellular molecular clock defects was accompanied by the demonstration that tissue-level rhythms in clock gene expression can also be recovered^16^. It remains unclear, however, as to whether SCN cellular synchrony is also elevated in this and other models. Similarly, while a previous report provided preliminary evidence that the disruptive effects of LL are somewhat reduced in *Vip*^*−/−*^ mice^39^, nothing has been reported of SCN function in this model under these conditions. Our current data demonstrate that the effect of an intercellular signaling deficit is diminished by exposure to LL, resulting in both improved wheel-running rhythms and increased SCN cellular synchrony. Interestingly, and consistent with our WT data in extended LL (>29 days), in animals with fully functional intra- and intercellular SCN processes, and indeed in some clock gene mutants with a less severe behavioral phenotype, exposure to LL is commonly associated with disruption of SCN molecular and neuronal function as well as the perturbation of other behavioral and physiological rhythms^26^,^40^,^41^,^42^,^43^,^44^,^45^,^46^,^47^,^48^,^49^. Thus, constant light is frequently detrimental to circadian rhythmicity in animals with either strong or fully functional circadian clocks, but can be beneficial to animals with a severely weakened circadian system. Moreover, in addition to the effects of LL on adult circadian systems, maintaining animals under LL during development can also influence subsequent circadian function; intact rats and mice housed under LL during development display reduced disruptions to rhythmicity when housed under LL as adults^50^,^51^ and mice deficient in expression of the core clock genes *Cry1* and *Cry2* (*Cry1*^*−/−*^*Cry2*^*−/−*^), when raised under LL conditions, express improved rhythmicity in constant darkness compared to LD-raised counterparts^52^. The developmental effects on behavioral and SCN rhythmicity and synchrony of raising neuropeptide deficient mice in LL is currently unknown, though Ono *et al.* (2013) report that, unlike *Vipr2*^*−/−*^ mice, housing adult LD-raised *Cry1*^*−/−*^*Cry2*^*−/−*^mice in LL does not improve circadian oscillations in locomotor activity.

Long-term exposure to LL resulted in a greater proportion of *Vipr2*^*−/−*^ mice expressing clear circadian rhythmicity than is seen in DD (current data as well as^24^,^32^,^53^), and notably, the rhythmicity of mice under DD conditions was not predicted by the responses of each individual to LL. Unlike scheduled voluntary exercise, whose rhythm promoting actions on wheel-running in neuropeptide signaling deficient mice are sustained for up to 4 weeks once the exercise regimen has terminated^53^, the rhythm-promoting actions of LL on behavior rapidly deteriorate on transfer to DD. This illustrates differences in the long-term reorganizing properties of different external stimuli on the circadian system of mice with intercellular signaling deficits^9^,^54^. These data indicate that continuous exposure to this signal is necessary to organize the *Vipr2*^*−/−*^ SCN to drive improved behavioral rhythmicity. However, initial exposure to constant light disrupted behavioral rhythms in *Vipr2*^*−/−*^ mice, which also indicates longer term adaptation and remodeling of the SCN to continual activation of light input pathways. Indeed, the stabilizing actions of extended LL on behavioral rhythms were robustly reproducible; re-exposure of *Vipr2*^*−/−*^ mice to LL for a second block of 36 days evoked the same behavioral parameters as had been observed in the first 36 day exposure, though interestingly, the ‘LL-like’ phenotype appeared to be more rapidly acquired during this second exposure, showing that although this phenotype is lost in DD some underlying changes at least partially persist.

There are several possible mechanisms underpinning the enhanced SCN cellular synchrony and improved rhythmic wheel-running arising from exposure to constant light. Electrophysiological investigations reveal that adult *Vipr2*^*−/−*^ SCN neurons tend to be hyperpolarized^55^ and are less spontaneously active than SCN neurons from adult C57BL/6 mice^56^. *Vipr2*^*−/−*^ SCN neurons retain acute responsiveness to glutamatergic signals^56^, such as those involved in transmitting photic information from the retina to the SCN, via the RHT^19^. Moreover, elevated electrical activity is sustained in adult *Vipr2*^*−/−*^ SCN *in* *vitro* in response to continuous glutamatergic stimulation over several hours^57^. As the RHT utilizes both glutamate and PACAP, it is likely that either elevated glutamatergic tone, or alterations in the glutamate-PACAP relationship, arising from exposure to constant light over several weeks, excites *Vipr2*^*−/−*^ neurons and improves their synchrony through stimulation of factor(s) that are independent of VIP-VPAC_2_ signaling. Candidates for this include: GABA, GRP and Neuromedin S, neurochemicals intrinsic to SCN neurons that have been implicated in cell-cell communication and SCN synchrony^6^,^32^,^57^,^58^; AVP, an abundant SCN-intrinsic neuropeptide^59^,^60^ with altered expression under LL^5^; and signaling via adenosine receptors, which are implicated in modifying the sensitivity of the SCN to retinal input^61^. Further, optic synapses in the SCN show plasticity^62^ and it is possible that constant light causes remodeling of synaptic contacts and gap junctions^63^ to improve SCN intercellular communication.

Outwith the direct effects of RHT signaling to the SCN, altered LL behavior of mice lacking 5-HT1a receptors suggests the potential for mechanistic involvement of arousal pathways in mediating the actions of LL on the circadian system^28^. Indeed, the possibility of increased activity of extra-SCN neural oscillators under LL has yet to be investigated and cannot be excluded^64^. Intriguingly, an as yet unidentified dopamine-sensitive extra-SCN oscillator, with the potential to explain a variety of non-canonical circadian phenomena, has recently been reported^65^,^66^. Interactions between such an oscillator and a weakened, but intact and functional SCN, such as that of *Vipr2*^*−/−*^ mice, provide a further possible avenue for investigation in the context of the current data.

In conclusion, our data illustrate that long-term exposure to LL promotes behavioral rhythmicity and SCN cellular synchrony in mice with deficient VIP-VPAC_2_ signaling. Further, we present evidence that, regardless of genotype, behavioral rhythmicity correlates closely with intercellular synchrony in the master circadian pacemaker but that, in WT SCN, this is associated with an increase in rhythmic cells, whilst in the *Vipr2*^*−/−*^ SCN, this is associated with reduced regional heterogeneity.

## Methods

### Animals

This study used adult male and female mice that expressed the *per1*::eGFP reporter and either expressed the *Vipr2* gene (WT) or lacked expression of the VPAC_2_ receptor (*Vipr2*^*−/−*^, see^7^). Prior to experimentation, all mice were group housed under a 12 h light:12 h dark (LD) cycle with *ad libitum* access to food (Beekay, B&K Universal, Hull, UK) and water. Environmental temperature was maintained at ∼23 °C and humidity at ∼40%. All procedures and experimental protocols were carried out in accordance with the UK Animals (Scientific Procedures) Act 1986 and approved by The University of Manchester Review Ethics Panel.

### Experimental Design and Behavioral Assessment

WT and *Vipr2*^*−/−*^ mice were individually housed in running wheel-equipped cages under a 12:12 LD cycle for 7–14 days (~3 × 10^14^ photons/s/cm^2^ lights on [~110 μW/cm^2^ lights on, from a broad spectrum fluorescent light source]) then released into LL (~3 × 10^14^ photons/s/cm^2^). Wheel revolutions were recorded in Chronobiology Kit (Stanford Software Systems, Santa Cruz, California, USA) using 5 min bins for analysis. Animals of each genotype were divided into 2 cohorts for assessment of 1) the effects of LL on wheel-running behavior (n = 12 WT, n = 24 *Vipr2*^*−/−*^) and 2) correlation of the effects of LL on wheel-running behavior with SCN *per1*::eGFP expression (n = 12 WT, n = 16 *Vipr2*^*−/−*^).

For experiment 1, mice were free-run undisturbed for 36 days of LL (LL1) during which behavioral rhythms were assessed in early (1-12 days), mid (13-24) and late LL (25-36). After LL1, n = 12 *Vipr2*^*−/−*^ mice were placed in DD for 36 days before a further 36 day LL epoch (LL2). The remaining *Vipr2*^*−/−*^ mice (n = 12) and all WT mice (n = 12) were removed from the experiment after LL1. Rhythmicity was assessed by 4 experienced observers, blind to genotype and experimental conditions, using a combined evaluation of locomotor actograms, periodograms, average waveforms and the time-frequency spectrogram obtained from sliding window fast Fourier transform (FFT)^34^, performed with a 7-day window that slid at 1-hr increments through wheel-running time series data smoothed with the Hodrick-Prescott filter (processed using Mathematica, Wolfram Research). In the majority of cases assessments of rhythmicity were unanimous (>90%), but for the infrequent cases where a consensus decision could not be easily reached, a final decision on rhythmicity was based on objective analysis of average waveforms. We determined that for a behavioral epoch to be considered rhythmic, a window centred on the most active part of the average waveform, consisting of 50% of its duration, should contain at least 75% of total activity. This method was validated on our entire data set and generated results entirely consistent with our prior assessments of behavior where we had initially been able to classify rhythmicity. For behavioral epochs that were clearly arrhythmic and for which no period could be determined from either periodogram analysis or visual inspection of actograms, a nominal value of 24 h was used to generate average waveforms. Where behavioral epochs were considered rhythmic, period was assessed in Chronobiology Kit using eyefit regression lines through onsets of activity and confirmed with spectral power analysis and *Chi*^*2*^ periodograms.

For experiment 2, mice were initially free-run in LL, as in experiment 1, though culled after increasing durations of LL ranging from a minimum of 22 days, to a maximum of 39 days. As the aim of this experiment was to investigate a possible association between time in LL, behavioral rhythmicity and SCN circadian function, mice were randomly assigned a cull timepoint in LL prior to the experiment commencing. This avoided the possibility of unintentional bias associated with selecting cull timepoints after behavioral data had already been collected. Immediately following cull, SCNs were collected for confocal imaging of *per1*::eGFP expression. Behavioral rhythms were analyzed for the last 10–14 days before cull and the behavior of all mice ranked in order of rhythmicity for correlation with SCN *per1*::eGFP data and duration of time in LL. Ranking was performed blind by 4 experienced observers and correlated strongly (*R*^2^ = 0.675; *p* < 0.0001) with the percentage of activity contained during a 50% window of the average waveform using the methods described above. Experimenters were blind to both the identity of mice in behavioral experiments and the parameters of SCN *per1*::eGFP data at the time of assessment for ranking of behavior. Behavior was further categorized as either rhythmic or arrhythmic (using the same procedure as in experiment 1) to make within-genotype comparisons of SCN *per1*::eGFP data according to behavioral rhythmicity.

### Confocal Imaging

Mice were culled by cervical dislocation following isoflurane anesthesia (Baxter Healthcare Ltd., Norfolk, UK), at circadian time (CT) 2–6 (to avoid different cull times influencing circadian parameters of SCN function^67^,^68^). Cultures were prepared as described previously^7^ using 250 μm thick SCN-containing coronal brain slices and 100 μg/ml and 25 μg/ml penicillin-streptomycin (Gibco Invitrogen Ltd, Paisley, UK) in collection medium and culture medium, respectively. Cultures were stored in darkness at 37 °C for ~24 h before imaging *per1*::eGFP fluorescence using a C1 confocal system running on a TE2000 inverted microscope (Nikon, Kingston, UK). Images were captured with a 10 x 0.3NA PlanFluotar objective (Nikon) and the system maintained at 37 °C. A 488 nm laser was used for excitation and emitted fluorescence detected using a 515/30 nm bandpass filter. 8 image ‘Z’ stacks were acquired every hour for ~120 h, using 4x Kalman averaging, 1.5x confocal zoom, an open pinhole and 0.2 Hz frame rate. Each stack covered a total Z depth of 32 μm and images were recorded at 512 × 512 pixels. Stacks were collapsed to an average projection using ImageJ and fluorescence profiles across time assessed for 30 individual cells selected at random^7^ using a region of interest tool. Raw fluorescence data were corrected for variations in background brightness by subtracting the brightness of a standardized, non-eGFP expressing, extra-SCN region from each data value before corrected data were smoothed using a 3 h running mean.

Cells were rated as rhythmic or arrhythmic by two experienced observers and amplitude, period and phase (time of peak at 12–36 hours) were assessed for each analyzed cell. The period of cellular rhythms was calculated using peak-peak and trough-trough durations for at least two cycles and phase data for individual cells were used to create Rayleigh plots to quantify the synchrony (phase-clustering) of rhythms between cells within each SCN^69^. Amplitude was calculated as the brightness differential from the peak used for phase analysis (12–36 hours into recording) to the following trough. Regional differences in period and phase within the SCN between behaviorally rhythmic and arrhythmic animals (period and phase heterogeneity) were assessed by classifying the location of each analyzed cell as either dorsal or ventral, based on anatomical characteristics of the SCN, and comparing the mean difference in each parameter between cells located in these two subregions.

### Statistics

As appropriate, statistically significant differences in continuous data were determined using either *t*-Tests (paired or unpaired) or one/two way ANOVA with *a priori* pairwise comparisons. Categorical data for the percentage of rhythmic and arrhythmic animals were analyzed using Fisher’s Exact Test with Freeman-Halton extension. Rayleigh Tests were used to determine statistical significance of cellular synchrony and the Rayleigh R statistic used to quantify the degree of synchrony. Statistical significances of correlations for ranked data were assessed using the non-parametric Spearman’s Rank Order Test. All statistical tests were run with α set at *p* < 0.05 required for significance, using Microsoft Excel, Graphpad Prism, the VasserStats online statistical resource (http://vassarstats.net/) and custom MATLAB scripts provided by Dr. Timothy Brown (University of Manchester).

## Additional Information

**How to cite this article**: Hughes, A. T.L. *et al.* Constant light enhances synchrony among circadian clock cells and promotes behavioral rhythms in VPAC_2_-signaling deficient mice. *Sci. Rep.* **5**, 14044; doi: 10.1038/srep14044 (2015).

## References

[b1] Mohawk J. A., Green C. B. & Takahashi J. S. Central and peripheral circadian clocks in mammals. Annu Rev Neurosci 35, 445–462 (2012). 22483041 10.1146/annurev-neuro-060909-153128 PMC3710582

[b2] Kuhlman S. J., Quintero J. E. & McMahon D. G. GFP fluorescence reports PeriodI circadian gene regulation in the mammalian biological clock. Neuroreport 11, 1479–1482 (2000). 10841361

[b3] Herzog E. D. Neurons and networks in daily rhythms. Nat Rev Neurosci 8, 790–802 (2007). 17882255 10.1038/nrn2215

[b4] Kallo I. *et al.* Transgenic approach reveals expression of the VPAC(2) receptor in phenotypically defined neurons in the mouse suprachiasmatic nucleus and in its efferent target sites. Eur J Neurosci 19, 2201–2211 (2004). 15090046 10.1111/j.0953-816X.2004.03335.x

[b5] An S., Tsai C., Ronecker J., Bayly A. & Herzog E. D. Spatiotemporal distribution of vasoactive intestinal polypeptide receptor 2 in mouse suprachiasmatic nucleus. J Comp Neurol 520, 2730–2741 (2012). 22684939 10.1002/cne.23078 PMC3961765

[b6] Maywood E. S. *et al.* Synchronization and maintenance of timekeeping in suprachiasmatic circadian clock cells by neuropeptidergic signaling. Curr Biol 16, 599–605 (2006). 16546085 10.1016/j.cub.2006.02.023

[b7] Hughes A. T. *et al.* Live imaging of altered period1 expression in the suprachiasmatic nuclei of Vipr2^−/−^ mice. J Neurochem 106, 1646–1657 (2008). 18554318 10.1111/j.1471-4159.2008.05520.x PMC2658715

[b8] Harmar A. J. *et al.* The VPAC(2) receptor is essential for circadian function in the mouse suprachiasmatic nuclei. Cell 109, 497–508 (2002). 12086606 10.1016/s0092-8674(02)00736-5

[b9] Lucassen E. A. *et al.* Role of vasoactive intestinal peptide in seasonal encoding by the suprachiasmatic nucleus clock. Eur J Neurosci 35, 1466–1474 (2012). 22512278 10.1111/j.1460-9568.2012.08054.x

[b10] Bechtold D. A., Brown T. M., Luckman S. M. & Piggins H. D. Metabolic rhythm abnormalities in mice lacking VIP-VPAC2 signaling. Am J Physiol Regul Integr Comp Physiol 294, R344–351 (2008). 18032467 10.1152/ajpregu.00667.2007

[b11] Schroeder A., Loh D. H., Jordan M. C., Roos K. P. & Colwell C. S. Circadian regulation of cardiovascular function: a role for vasoactive intestinal peptide. Am J Physiol Heart Circ Physiol 300, H241–250 (2011). 20952671 10.1152/ajpheart.00190.2010 PMC4116412

[b12] Reppert S. M. & Weaver D. R. Coordination of circadian timing in mammals. Nature 418, 935–941 (2002). 12198538 10.1038/nature00965

[b13] Bae K. *et al.* Differential functions of mPer1, mPer2, and mPer3 in the SCN circadian clock. Neuron 30, 525–536 (2001). 11395012 10.1016/s0896-6273(01)00302-6

[b14] Yoo S. H. *et al.* A noncanonical E-box enhancer drives mouse Period2 circadian oscillations *in vivo*. Proc Natl Acad Sci USA 102, 2608–2613 (2005). 15699353 10.1073/pnas.0409763102 PMC548324

[b15] Zheng B. *et al.* Nonredundant roles of the mPer1 and mPer2 genes in the mammalian circadian clock. Cell 105, 683–694 (2001). 11389837 10.1016/s0092-8674(01)00380-4

[b16] Abraham D. *et al.* Restoration of circadian rhythmicity in circadian clock-deficient mice in constant light. J Biol Rhythms 21, 169–176 (2006). 16731656 10.1177/0748730406288040

[b17] Spoelstra K. & Daan S. Effects of constant light on circadian rhythmicity in mice lacking functional cry genes: dissimilar from per mutants. J Comp Physiol A Neuroethol Sens Neural Behav Physiol 194, 235–242 (2008). 18057941 10.1007/s00359-007-0301-3

[b18] Steinlechner S. *et al.* Robust circadian rhythmicity of Per1 and Per2 mutant mice in constant light, and dynamics of Per1 and Per2 gene expression under long and short photoperiods. J Biol Rhythms 17, 202–209 (2002). 12054191 10.1177/074873040201700303

[b19] Ebling F. J. The role of glutamate in the photic regulation of the suprachiasmatic nucleus. Prog Neurobiol 50, 109–132 (1996). 8971980 10.1016/s0301-0082(96)00032-9

[b20] Hannibal J. Roles of PACAP-containing retinal ganglion cells in circadian timing. Int Rev Cytol 251, 1–39 (2006). 16939776 10.1016/S0074-7696(06)51001-0

[b21] Albrecht U., Zheng B., Larkin D., Sun Z. S. & Lee C. C. MPer1 and mper2 are essential for normal resetting of the circadian clock. J Biol Rhythms 16, 100–104 (2001). 11302552 10.1177/074873001129001791

[b22] Dragich J. M. *et al.* The role of the neuropeptides PACAP and VIP in the photic regulation of gene expression in the suprachiasmatic nucleus. Eur J Neurosci 31, 864–875 (2010). 20180841 10.1111/j.1460-9568.2010.07119.x

[b23] Hughes A. T., Fahey B., Cutler D. J., Coogan A. N. & Piggins H. D. Aberrant gating of photic input to the suprachiasmatic circadian pacemaker of mice lacking the VPAC2 receptor. J Neurosci 24, 3522–3526 (2004). 15071099 10.1523/JNEUROSCI.5345-03.2004 PMC6729740

[b24] Hughes A. T. & Piggins H. D. Behavioral responses of Vipr2^−/−^ mice to light. J Biol Rhythms 23, 211–219 (2008). 18487413 10.1177/0748730408316290

[b25] Maywood E. S., O’Neill J. S., Chesham J. E. & Hastings M. H. Minireview: The circadian clockwork of the suprachiasmatic nuclei—analysis of a cellular oscillator that drives endocrine rhythms. Endocrinology 148, 5624–5634 (2007). 17901233 10.1210/en.2007-0660

[b26] Marston O. J. *et al.* Circadian and dark-pulse activation of orexin/hypocretin neurons. Mol Brain 1, 19 (2008). 19055781 10.1186/1756-6606-1-19 PMC2632999

[b27] Ohta H., Yamazaki S. & McMahon D. G. Constant light desynchronizes mammalian clock neurons. Nature Neuroscience 8, 267–269 (2005). 15746913 10.1038/nn1395

[b28] Smith V. M. *et al.* Effects of lighting condition on circadian behavior in 5-HT1A receptor knockout mice. Physiol Behav 139, 136–144 (2015). 25446224 10.1016/j.physbeh.2014.11.005

[b29] Schwartz W. J. & Zimmerman P. Circadian Timekeeping in Balb/C and C57bl/6 Inbred Mouse Strains. J Neurosci 10, 3685–3694 (1990). 2230953 10.1523/JNEUROSCI.10-11-03685.1990 PMC6570095

[b30] Pittendrigh C. S. & Daan S. Functional-Analysis of Circadian Pacemakers in Nocturnal Rodents .1. Stability and Lability of Spontaneous Frequency. Journal of Comparative Physiology 106, 223–252 (1976).

[b31] LeSauter J. *et al.* A short half-life GFP mouse model for analysis of suprachiasmatic nucleus organization. Brain Research 964, 279–287 (2003). 12576188 10.1016/s0006-8993(02)04084-2 PMC3271845

[b32] Aton S. J., Colwell C. S., Harmar A. J., Waschek J. & Herzog E. D. Vasoactive intestinal polypeptide mediates circadian rhythmicity and synchrony in mammalian clock neurons. Nat Neurosci 8, 476–483 (2005). 15750589 10.1038/nn1419 PMC1628303

[b33] Hannibal J., Hsiung H. M. & Fahrenkrug J. Temporal phasing of locomotor activity, heart rate rhythmicity, and core body temperature is disrupted in VIP receptor 2-deficient mice. Am J Physiol Regul Integr Comp Physiol 300, R519–530 (2011). 21178124 10.1152/ajpregu.00599.2010

[b34] Myung J. *et al.* Period coding of Bmal1 oscillators in the suprachiasmatic nucleus. J Neurosci 32, 8900–8918 (2012). 22745491 10.1523/JNEUROSCI.5586-11.2012 PMC6622328

[b35] Pauls S. *et al.* Differential contributions of intra-cellular and inter-cellular mechanisms to the spatial and temporal architecture of the suprachiasmatic nucleus circadian circuitry in wild-type, cryptochrome-null and vasoactive intestinal peptide receptor 2-null mutant mice. Eur J Neurosci 40, 2528–2540 (2014). 24891292 10.1111/ejn.12631 PMC4159586

[b36] Foley N. C. *et al.* Characterization of orderly spatiotemporal patterns of clock gene activation in mammalian suprachiasmatic nucleus. Eur J Neurosci 33, 1851–1865 (2011). 21488990 10.1111/j.1460-9568.2011.07682.x PMC3423955

[b37] Evans J. A., Leise T. L., Castanon-Cervantes O. & Davidson A. J. Intrinsic regulation of spatiotemporal organization within the suprachiasmatic nucleus. PLoS One 6, e15869, 10.1371/journal.pone.0015869 (2011). 21249213 PMC3017566

[b38] Myung J. *et al.* GABA-mediated repulsive coupling between circadian clock neurons in the SCN encodes seasonal time. Proc Natl Acad Sci USA 112, E3920–E3929 (2015). 26130804 10.1073/pnas.1421200112 PMC4517217

[b39] An S. *et al.* A neuropeptide speeds circadian entrainment by reducing intercellular synchrony. Proc Natl Acad Sci USA 110, E4355–4361 (2013). 24167276 10.1073/pnas.1307088110 PMC3832006

[b40] Evans J. A., Pan H., Liu A. C. & Welsh D. K. Cry1^−/−^ circadian rhythmicity depends on SCN intercellular coupling. J Biol Rhythms 27, 443–452 (2012). 23223370 10.1177/0748730412461246 PMC3578226

[b41] Munoz M., Peirson S. N., Hankins M. W. & Foster R. G. Long-term constant light induces constitutive elevated expression of mPER2 protein in the murine SCN: a molecular basis for Aschoff’s rule? J Biol Rhythms 20, 3–14 (2005). 15654066 10.1177/0748730404272858

[b42] Coomans C. P. *et al.* Detrimental effects of constant light exposure and high-fat diet on circadian energy metabolism and insulin sensitivity. Faseb J 27, 1721–1732 (2013). 23303208 10.1096/fj.12-210898

[b43] Eastman C. & Rechtschaffen A. Circadian temperature and wake rhythms of rats exposed to prolonged continuous illumination. Physiol Behav 31, 417–427 (1983). 6657763 10.1016/0031-9384(83)90061-6

[b44] Waite E. J. *et al.* Ultradian corticosterone secretion is maintained in the absence of circadian cues. Eur J Neurosci 36, 3142–3150 (2012). 22823558 10.1111/j.1460-9568.2012.08213.x

[b45] Dallmann R., DeBruyne J. P. & Weaver D. R. Photic resetting and entrainment in CLOCK-deficient mice. J Biol Rhythms 26, 390–401 (2011). 21921293 10.1177/0748730411414345 PMC3437920

[b46] Sudo M. *et al.* Constant light housing attenuates circadian rhythms of mPer2 mRNA and mPER2 protein expression in the suprachiasmatic nucleus of mice. Neuroscience 121, 493–499 (2003). 14522008 10.1016/s0306-4522(03)00457-3

[b47] Tapia-Osorio A., Salgado-Delgado R., Angeles-Castellanos M. & Escobar C. Disruption of circadian rhythms due to chronic constant light leads to depressive and anxiety-like behaviors in the rat. Behav Brain Res 252, 1–9 (2013). 23714074 10.1016/j.bbr.2013.05.028

[b48] Park S. Y. *et al.* Constant light disrupts the circadian rhythm of steroidogenic proteins in the rat adrenal gland. Mol Cell Endocrinol 371, 114–123 (2013). 23178164 10.1016/j.mce.2012.11.010

[b49] Honma K. I. & Hiroshige T. Endogenous ultradian rhythms in rats exposed to prolonged continuous light. Am J Physiol 235, R250–256 (1978). 727287 10.1152/ajpregu.1978.235.5.R250

[b50] Canal-Corretger M. M., Cambras T., Vilaplana J. & Diez-Noguera A. Bright light during lactation alters the functioning of the circadian system of adult rats. Am J Physiol Regul Integr Comp Physiol 278, R201–208 (2000). 10644640 10.1152/ajpregu.2000.278.1.R201

[b51] Brooks E., Patel D. & Canal M. M. Programming of mice circadian photic responses by postnatal light environment. PLoS One 9, e97160, 10.1371/journal.pone.0097160PONE-D-14-03060 [pii] (2014). 24842115 PMC4026311

[b52] Ono D., Honma S. & Honma K. Postnatal constant light compensates Cryptochrome1 and 2 double deficiency for disruption of circadian behavioral rhythms in mice under constant dark. PLoS One 8, e80615, 10.1371/journal.pone.0080615PONE-D-13-31531 [pii] (2013). 24278295 PMC3835422

[b53] Power A., Hughes A. T., Samuels R. E. & Piggins H. D. Rhythm-promoting actions of exercise in mice with deficient neuropeptide signaling. J Biol Rhythms 25, 235–246 (2010). 20679493 10.1177/0748730410374446

[b54] Hughes A. T. & Piggins H. D. Feedback actions of locomotor activity to the circadian clock. Prog Brain Res 199, 305–336 (2012). 22877673 10.1016/B978-0-444-59427-3.00018-6

[b55] Pakhotin P., Harmar A. J., Verkhratsky A. & Piggins H. VIP receptors control excitability of suprachiasmatic nuclei neurones. Pflugers Arch 452, 7–15 (2006). 16283205 10.1007/s00424-005-0003-z

[b56] Cutler D. J. *et al.* The mouse VPAC(2) receptor confers suprachiasmatic nuclei cellular rhythmicity and responsiveness to vasoactive intestinal polypeptide *in vitro*. Eur J Neurosci 17, 197–204 (2003). 12542655 10.1046/j.1460-9568.2003.02425.x

[b57] Brown T. M., Hughes A. T. & Piggins H. D. Gastrin-releasing peptide promotes suprachiasmatic nuclei cellular rhythmicity in the absence of vasoactive intestinal polypeptide-VPAC2 receptor signaling. J Neurosci 25, 11155–11164 (2005). 16319315 10.1523/JNEUROSCI.3821-05.2005 PMC6725650

[b58] Lee I. T. *et al.* Neuromedin S-producing neurons act as essential pacemakers in the suprachiasmatic nucleus to couple clock neurons and dictate circadian rhythms. Neuron 85, 1086–1102 (2015). 25741729 10.1016/j.neuron.2015.02.006 PMC5811223

[b59] Yamaguchi Y. *et al.* Mice genetically deficient in vasopressin V1a and V1b receptors are resistant to jet lag. Science 342, 85–90 (2013). 24092737 10.1126/science.1238599

[b60] Mieda M. *et al.* Cellular clocks in AVP neurons of the SCN are critical for intercellular coupling regulating circadian behavior rhythm. Neuron 85, 1103–1116 (2015). 25741730 10.1016/j.neuron.2015.02.005

[b61] van Diepen H. C. *et al.* Caffeine increases light responsiveness of the mouse circadian pacemaker. Eur J Neurosci 40, 3504–3511 (2014). 25196050 10.1111/ejn.12715

[b62] Guldner F. H., Bahar E., Young C. A. & Ingham C. A. Structural plasticity of optic synapses in the rat suprachiasmatic nucleus: adaptation to long-term influence of light and darkness. Cell Tissue Res 287, 43–60 (1997). 9011401 10.1007/s004410050730

[b63] Long M. A., Jutras M. J., Connors B. W. & Burwell R. D. Electrical synapses coordinate activity in the suprachiasmatic nucleus. Nat Neurosci 8, 61–66 (2005). 15580271 10.1038/nn1361

[b64] Guilding C. & Piggins H. D. Challenging the omnipotence of the suprachiasmatic timekeeper: are circadian oscillators present throughout the mammalian brain? Eur J Neurosci 25, 3195–3216 (2007). 17552989 10.1111/j.1460-9568.2007.05581.x

[b65] Blum I. D. *et al.* A highly tunable dopaminergic oscillator generates ultradian rhythms of behavioral arousal. Elife 3, 10.7554/eLife.05105 (2014). PMC4337656 25546305

[b66] Steele A. D. & Mistlberger R. E. Activity is a slave to many masters. Elife 4, 10.7554/eLife.06351 (2014). PMC4337654 25675211

[b67] Hughes A. T., Guilding C. & Piggins H. D. Neuropeptide signaling differentially affects phase maintenance and rhythm generation in SCN and extra-SCN circadian oscillators. PLoS One 6, e18926, 10.1371/journal.PONE.0018926 (2011). 21559484 PMC3084722

[b68] Yoshikawa T., Yamazaki S. & Menaker M. Effects of preparation time on phase of cultured tissues reveal complexity of circadian organization. J Biol Rhythms 20, 500–512 (2005). 16275769 10.1177/0748730405280775 PMC1470468

[b69] Herzog E. D., Kiss I. Z. & Mazuski C. Measuring synchrony in the Mammalian central circadian circuit. Methods Enzymol 552, 3–22 (2015). 25707270 10.1016/bs.mie.2014.10.042 PMC5110928

